# Garcinielliptone G from *Garcinia subelliptica* Induces Apoptosis in Acute Leukemia Cells

**DOI:** 10.3390/molecules26092422

**Published:** 2021-04-21

**Authors:** YoungSook Yun, Mariko Shioura, Yukio Hitotsuyanagi, Satoshi Yotsumoto, Yuji Takahashi, Yutaka Aoyagi, Takeshi Kinoshita, Koichi Takeya, Hideshi Inoue

**Affiliations:** 1 School of Life Sciences, Tokyo University of Pharmacy and Life Sciences, 1432-1 Horinouchi, Hachioji, Tokyo 192-0392, Japan; salt880822@gmail.com (M.S.); yotumoto@toyaku.ac.jp (S.Y.); yuji@toyaku.ac.jp (Y.T.); hinoue@toyaku.ac.jp (H.I.); 2 School of Pharmacy, Tokyo University of Pharmacy and Life Sciences, 1432-1 Horinouchi, Hachioji, Tokyo 192-0392, Japan; yukioh@toyaku.ac.jp (Y.H.); yutaka@kinjo-u.ac.jp (Y.A.); takeya0520@nifty.com (K.T.); 3 Faculty of Pharma-Science, Teikyo University, 2-11-1 Kaga, Itabashi, Tokyo 173-8605, Japan; kinoshita19480414@gmail.com

**Keywords:** *Garcinia subelliptica*, garcinielliptone G, apoptosis, human acute leukemia cell lines

## Abstract

Cytotoxicity and apoptosis-inducing properties of compounds isolated from *Garcinia subelliptica* leaves were investigated. The hexane-soluble portion of MeOH extracts of *G. subelliptica* leaves that showed cytotoxic activity was separated to yield seven compounds **1**–**7**. Chemical structure analysis using NMR spectroscopy and mass spectrometry confirmed that compound **1** was canophyllol, and compounds **2**–**7** were garcinielliptones N, O, J, G, F, and garcinielliptin oxide, respectively. Among them, garcinielliptone G (**5**) showed growth inhibition by causing apoptosis in THP-1 and Jurkat cells derived from human acute monocytic leukemia and T lymphocyte cells, respectively. Apoptosis induced by garcinielliptone G (**5**) was demonstrated by the detection of early apoptotic cells with fluorescein-labeled Annexin V and increases in cleaved caspase-3 and cleaved PARP protein levels. However, the addition of caspase inhibitor Z-VAD-FMK did not affect growth arrest or apoptosis induction. These results suggest that garcinielliptone G (**5**) can induce both caspase-3 activation and caspase-independent apoptosis. Therefore, garcinielliptone G (**5**) may be a potential candidate for acute leukemia treatment.

## 1. Introduction

Leukemia is a group of heterogeneous hematopoietic stem cell malignancies. It is characterized by abnormal accumulation of undifferentiated blasts capable of uncontrolled proliferation in the bone marrow, preventing normal blood cell production. Acute leukemia (AL) is a common malignancy among children and adults worldwide and is one of the most common deadly cancers [1]. AL includes acute myeloid leukemia and acute lymphoblastic leukemia. The application of risk-adapted therapy and improved supportive care have increased 5-year survival rates, however, relapse rates have not significantly changed. In addition, conventional leukemia therapies have side effects, such as hepatotoxicity and myelosuppression [2].

Medicinal plants have historically proven their value as a source of molecules with therapeutic potential, and remain a valuable resource for the development of novel drugs [3] that can be applied to reduce the proliferation and metastasis of cancer cells. Plant-derived compounds may be used alone [4] or in combination with known chemotherapeutic agents [5,6,7].

The *Garcinia* species have been traditionally used to treat inflammatory diseases in Southeast Asian countries [8]. Ethanol and dichloromethane extracts of the fruit hull of *G. mangostana* have anti-inflammatory activity [9], and the ethyl acetate extract of *G. hanburyi* has anti-inflammatory, analgesic, and antipyretic properties [10]. In addition, hydroalcoholic extracts of *G. gardneriana* leaves show anti-inflammatory activity in several experimental models [11].

*G. subelliptica* Merr. (Clusiaceae) is a windbreak tree whose bark and leaves are used for dyes in Amami Oshima and Okinawa, Japan. As its constituents, benzophenones and xanthones have been isolated from its fruits, seeds, wood, bark, and roots.

In addition, biflavonoids have been isolated from its leaves, and triterpenoids have been isolated from its leaves and fruits. Several compounds have been demonstrated to show antioxidant, anti-inflammatory, and cytotoxic activities in some human cancer cell lines [12]. In this study, we screened a collection of plant extracts native to Iriomote Island, Japan, and found that methanol extracts of *G. subelliptica* leaves were cytotoxic against both human acute monocytic leukemia THP-1 cells and human acute T cell leukemia Jurkat cells. Seven compounds were isolated by bioassay-guided fractionation of the extracts and subsequent separation of the active fractions, and one of them exhibited cytotoxic and apoptosis-inducing effects on THP-1 and Jurkat cells. This paper reports isolation of the cytotoxic principle from *G. subelliptica* leaves and its apoptotic effects on THP-1 and Jurkat cells.

## 2. Results

### 2.1. Purification and Identification of Compounds from G. subelliptica Leaves

**Figure 1:**
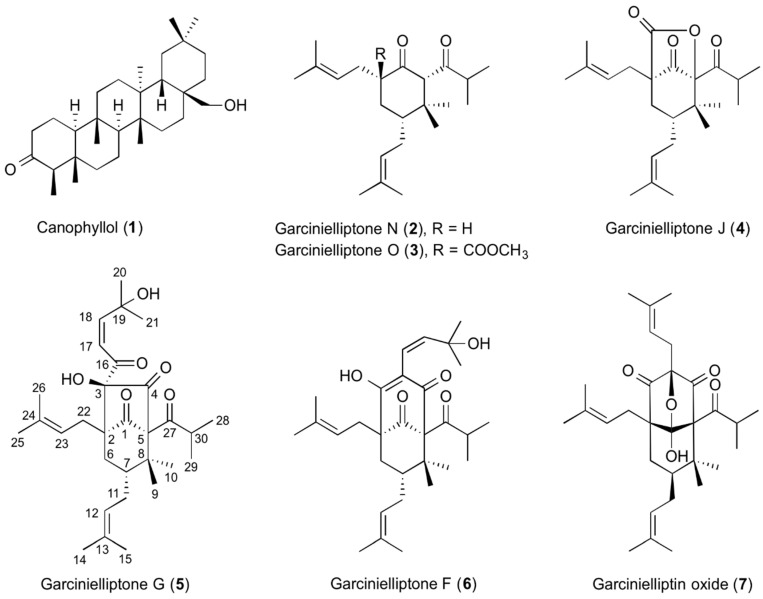
Compounds isolated from *Garcinia subelliptica* leaves.

Methanolic extracts of *G. subelliptica* leaves were found to be cytotoxic by the WST-1 assay (Figure S1a). Extracts were suspended in water and sequentially partitioned with *n*-hexane, ethyl acetate, and *n*-butanol. The *n*-hexane-soluble portion showed cytotoxic activity (Figure S1a) and was further subjected to HP-20 column chromatography to yield six fractions (Frs. H-I to H-VI). Cytotoxicity was found in Frs. H-IV, H-V, and H-VI (Figure S1b). Fr. H-V was then further separated by HPLC to give seven compounds. The isolated compounds were identified as canophyllol (**1**) [13], garcinielliptone N (**2**) [14], garcinielliptone O (**3**) [14], garcinielliptone J (**4**) [15], garcinielliptone G (**5**) [15], garcinielliptone F (**6**) [15], and garcinielliptin oxide (**7**) [16,17] by comparing their NMR and MS spectra with those in the literature (Figure 1 and Figures S4–S10). Compound **1** was a friedelin-type triterpenoid and compounds **2**–**7** contained the polyprenylated cyclohexanone structure.

### 2.2. Inhibition of Cell Growth in THP-1 and Jurkat Cells

**Figure 2:**
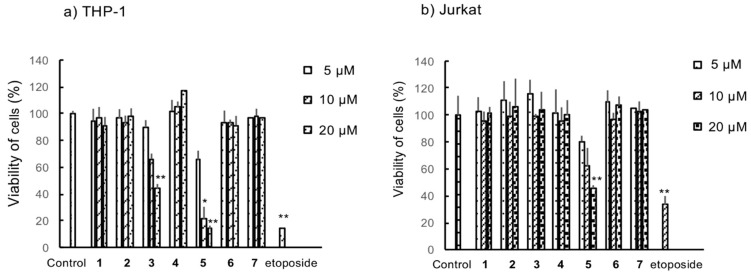
Cytotoxicity of compounds (**1**–**7**) in (**a**) THP-1 and (**b**) Jurkat cells. Cells were treated with the indicated concentrations of compounds for 24 h. Etoposide (1 µM) was used as a positive control. Cell viability was determined by the WST-1 assay. Values are presented as the mean ± SD of three independent experiments. * *p* < 0.05, ** *p* < 0.01.

Compounds **1**–**7** were evaluated for cytotoxicity against THP-1 and Jurkat cells using the WST-1 assay. As presented in Figure 2, only garcinielliptone G (**5**) exhibited cell growth inhibition in both THP-1 and Jurkat cells in a concentration-dependent manner. Garcinielliptone O (**3**) exhibited significant cytotoxicity only in THP-1 cells at a concentration of 20 µM.

### 2.3. Apoptosis Induction by Garcinielliptone G (**5**)

**Figure 3:**
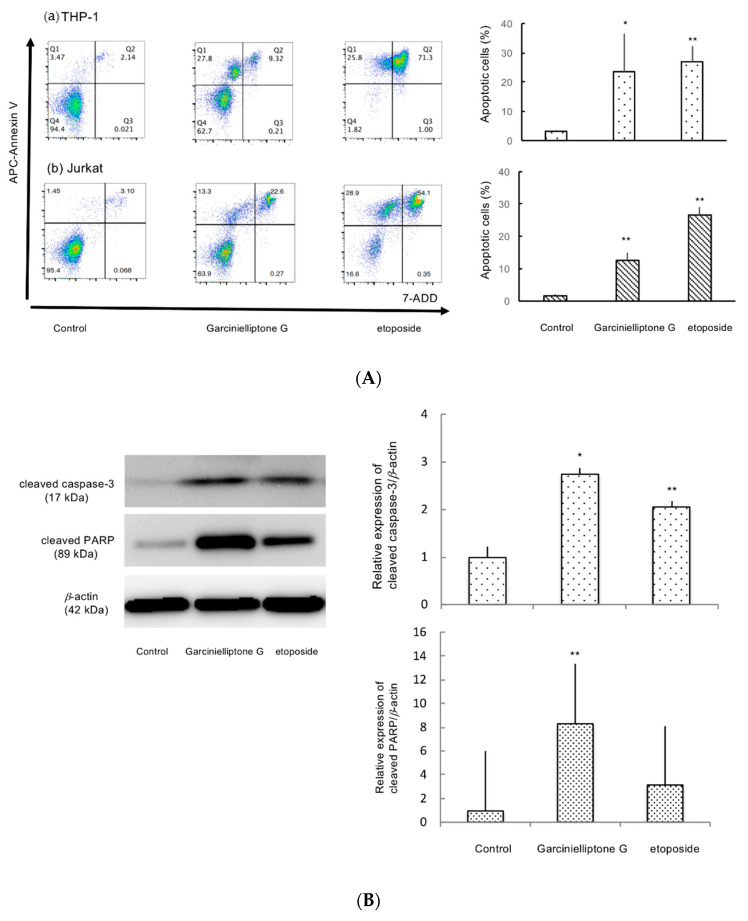

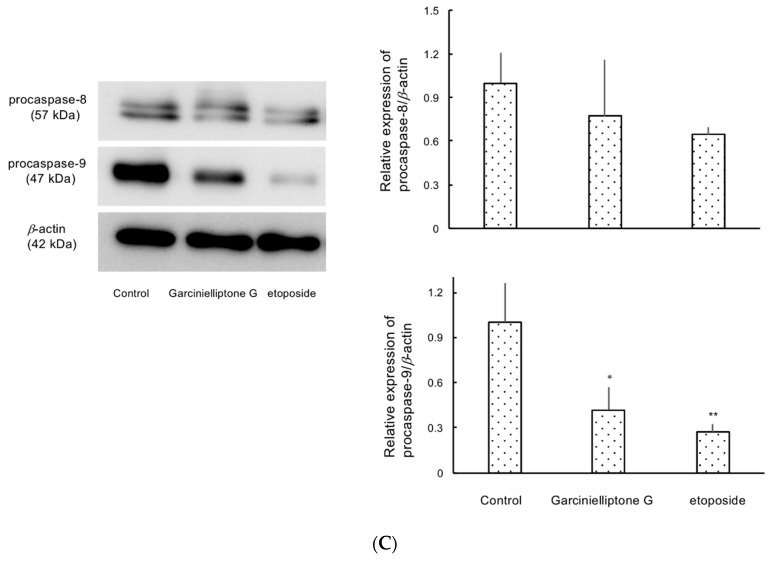
Garcinielliptone G (**5**) induces apoptosis. (**A**) Cells were treated with 10 µM garcinielliptone G (**5**) for 24 h, stained with APC Annexin/7-ADD, and analyzed by flow cytometry. Etoposide (1 µM) was used as a positive control. (**B**) and (**C**) THP-1 cells were treated with 10 µM garcinielliptone G (**5**) for 24 h, and cell lysates were prepared. Cleaved caspase 3, cleaved PARP, procaspase 8, and procaspase 9 protein levels were determined by western blot analysis. *β*-actin expression was determined to confirm equal protein loading. Values are presented as the mean ± SD of three independent experiments. * *p* < 0.05, ** *p* < 0.01.

To examine the type of cell death observed, cells were treated with garcinielliptone G (**5**) for 24 h and subjected to fluorescence-activated cell sorting analysis. During the early stage of apoptosis, externalization of phosphatidyl serine occurs, which can be visualized by binding to APC Annexin V. As presented in Figure 3A, the percentage of cells in the early stage of apoptosis was about 28% and 13% for THP-1 and Jurkat cells, respectively. Apoptosis induction was also confirmed by the observation that cells treated with garcinielliptone G (**5**) for 24 h were labeled with Annexin V-FITC (Figure S2). Most apoptotic signaling pathways are controlled by caspases that belong to a group of cysteine proteases, one of whose substrates is poly (ADP-ribose) polymerase (PARP) [18]. Cleaved caspase-3 (17 kDa) and cleaved PARP (85 kDa) protein levels were increased in THP-1 cells after treatment with garcinielliptone G (**5**) (Figure 3B). These results also suggest that apoptosis occurred in THP-1 cells. Two pathways are known to initiate apoptosis, and caspase-8 and caspase-9 are the major initiators for the extrinsic and intrinsic caspase cascades, respectively [19]. As presented in Figure 3C, garcinielliptone G (**5**) treatment significantly reduced procaspase-9 protein levels. Garcinielliptone G (**5**) treatment also reduced procaspase-8 protein levels, but this difference was not statistically significant. These data suggest that garcinielliptone G (**5**) induces apoptosis via the mitochondria-dependent intrinsic pathway and potentially other pathways.

### 2.4. Caspase-Independent Apoptosis Induction by Garcinielliptone G (**5**)

**Figure 4:**
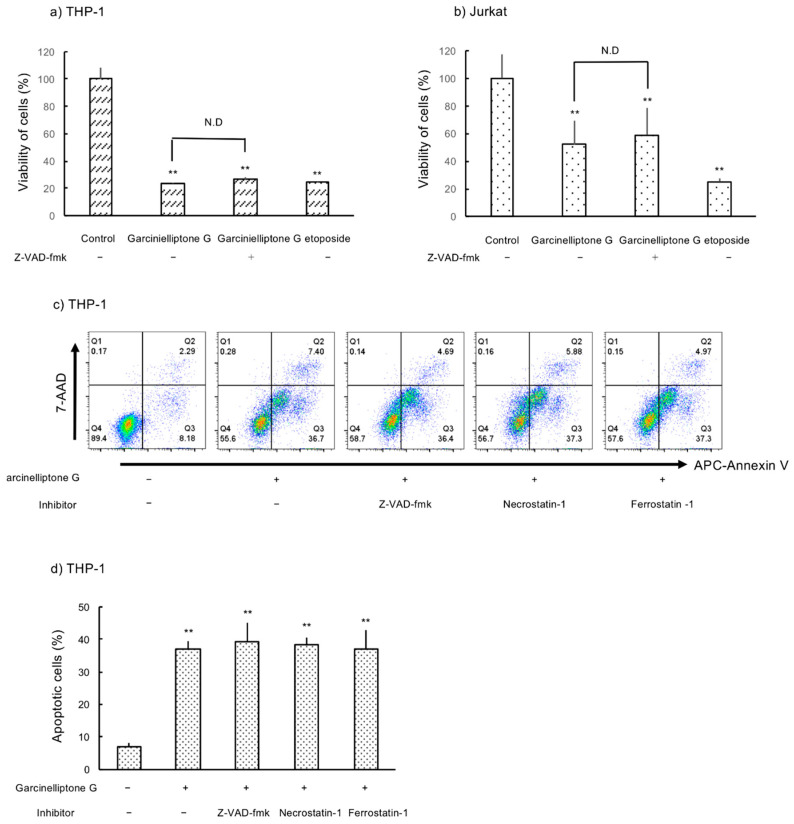
Pan-caspase inhibitor Z-VAD-FMK does not prevent garcinielliptone G (**5**)-induced apoptosis. (**a**) THP-1 and (**b**) Jurkat cells were treated with Z-VAD-FMK (50 µM) 1 h before garcinielliptone G (**5**) addition, and cell viability was determined by the WST-1 assay. (**c**) and (**d**) THP-1 cells were treated with inhibitors Z-VAD-FMK (50 µM), necrostatin-1 (10 µM), and ferrostatin-1 (1 µM) 1 h prior to the addition of garcinielliptone G (5). These inhibitors did not prevent apoptosis caused by garcinielliptone G (**5**). Values are presented as the mean ± SD of three independent experiments. * *p* < 0.05, ** *p* < 0.01.

To determine whether apoptosis caused by garcinielliptone G (**5**) is mediated by caspases, THP-1 and Jurkat cells were treated with pan-caspase inhibitor, Z-VAD-FMK (50 µM), 1 h prior to garcinielliptone G (**5**) treatment. Cell viability was analyzed using the WST-1 assay after 4 h treatment with garcinielliptone G (**5**). As shown in Figure 4a,b, the inhibitor did not recover THP-1 or Jurkat cell viability. The ratio of apoptotic THP-1 cells shown by labeling with APC Annexin V under garcinielliptone G (**5**) treatment was also unaffected by Z-VAD-FMK. These findings suggest that apoptosis caused by garcinielliptone G (**5**) was also induced through a caspase-independent pathway. Treatment of THP-1 cells with necrostatin-1 (10 µM) or ferrostatin-1 (1 µM), necroptosis and ferroptosis inhibitors, respectively, did not decrease the number of cells stained with APC Annexin V (Figure 4c,d). This suggests that caspase-dependent apoptosis, necroptosis, and ferroptosis are not essential for garcinielliptone G (**5**)-induced apoptosis in THP-1 cells.

## 3. Discussion

Antioxidant xanthones have been isolated from *G. subelliptica* wood [20] and root bark [21], and prenylated acylphloroglucinols have been isolated from its seeds. However, there are few reports on the phytochemical investigation of *G. subelliptica* leaves [22], and only isoprenylated biflavonoids have been isolated from the leaves. Although compounds **2**–**7** have already been isolated from *G. subelliptica* seeds, they were isolated from the leaves of this plant species for the first time in the present study. Cytotoxic benzylphloroglucinol including garcinielliptone FB [23] and garcinielliptones A and B [24] were isolated from *G. subelliptica* fruits or pericarp, and it was suggested that the benzoyl group in the phloroglucinol skeleton plays a crucial role in its cytotoxic activity [24]. Comparison of the activity of compounds **2**–**4**, **6**, and **7** suggests that cell death induced by garcinielliptone G (**5**) may be related to its hydroxyl group and 4-hydroxy-4-methyl-2-pentenoyl group at position 3. The increase in cleaved PARP and cleaved caspase-3 protein levels or number of cells labeled with APC Annexin V by garcinielliptone G (**5**) treatment demonstrated that garcinielliptone G (**5**) induces cell apoptosis. Furthermore, because Z-VAD-FMK treatment did not decrease the number of apoptotic cells, this caspase-independent apoptosis was probably induced by garcinielliptone G (**5**). EndoG is a mitochondrial protein that is released from mitochondria followed by nuclear translocation during the caspase-independent apoptotic process [25]. To investigate whether EndoG is involved in garcinielliptone G (**5**)-induced apoptosis in THP-1 cells, EndoG levels in nuclear and cytosolic fractions including mitochondria were examined by Western blotting. As shown in Figure S3, EndoG was decreased in the cytosolic fraction and increased in the nuclear fraction after garcinielliptone G (**5**) stimulation. This result suggests that garcinielliptone G (**5**)-induced apoptosis might occur through the EndoG-mediated pathway.

## 4. Materials and Method

### 4.1. Plant Materials

*G. subelliptica* leaves were collected at Iriomote Island, Okinawa Prefecture, Japan. The plant material was identified by one of authors (T.K.), and a voucher specimen was deposited at the Laboratory of Molecular and Chemical Biology, School of Life Sciences, Tokyo University of Pharmacy and Life Sciences (Gs-2008).

### 4.2. Reagents

RPMI 1640 powder was purchased from Nissui Pharmaceutical (Tokyo, Japan), and l-glutamine was purchased from Wako Pure Chemical Industries (Osaka, Japan). Allophycocyanin-coupled (APC) Annexin V/*7*-Amino-actinomycin D (7-AAD) kit was purchased from BioLegend (San Diego, CA, USA). Annexin V- fluorescein isothiocyanate (FITC) was purchased from MBL (Nagoya, Japan), and Hoechst 33342 was purchased from Dojindo (Rockville, MD, USA). Necrostatin-1 and ferrostatin-1 were purchased from Cayman Chemical Company (Ann Arbor, MI, USA), and WST-1 was purchased from Roche (Basel, Switzerland). Z-Val-Ala-Asp-fluoromethylketone (Z-VAD-FMK) was purchased from Cell Signaling Technology (Danvers, MA, USA). All organic solvents were purchased from Kanto Chemicals (Tokyo, Japan).

### 4.3. Instrumentation

NMR spectra were measured using a DRX-500 or DPX-400 spectrometer (Bruker, Karlsruhe, Germany) at 300 K. ^1^H-NMR chemical shifts for CDCl_3_ were calibrated to residual CHCl_3_ resonances at 7.26 ppm, and ^13^C-NMR chemical shifts were calibrated to solvent peaks at 77.0 ppm. Mass spectra were obtained using a Micromass spectrometer (Manchester, UK). Preparative HPLC was carried out using a reversed-phase column, Inertsil PREP-ODS (10 µM, 20 mm × 250 mm, GL Sciences, Tokyo, Japan) on an LC-10AS pump equipped with a SPD-20A UV/VIS detector (Shimadzu, Kyoto, Japan), and a PU-986 pump with a UV-970 detector (JASCO, Tokyo, Japan) at a flow rate of 5 mL/min. High-content imaging of cells was performed using an Operetta CLS (PerkinElmer, Waltham, MA, USA). For the WST-1 assay, absorbance was measured using a Corona multimode microplate reader (Corona Electric, Ibaraki, Japan). Flow cytometry was performed using FACSVerse (BD Biosciences, Franklin Lake, NJ, USA).

### 4.4. Extraction, Isolation and Purification of Compounds from G. subelliptica Leaves

Dried *G. subelliptica* leaves (1.72 kg) were extracted with methanol (28 L) at 40 °C. After filtration, the solvent was evaporated under reduced pressure. The methanolic residue (297 g) was suspended in water and then extracted successively with *n*-hexane, ethyl acetate, and *n*-butanol. Each extract and the water residue were concentrated and dried in vacuo to give *n*-hexane (104.5 g), ethyl acetate (77.2 g), and *n*-butanol (95.3 g) extracts and a water-soluble portion. The *n*-hexane extract was loaded into an HP-20 column and eluted sequentially with methanol/water mixtures (3:7, 1:1, 6.5:3.5, 8:2, and 0:1, *v*/*v*, 10 L) and then ethyl acetate (15 L) to yield six fractions (Frs. H-I to H-VI). Each fraction was evaporated to dryness. As the fifth fraction, Fr. H-V, was the most cytotoxic, this fraction was further separated by silica gel chromatography using a stepwise gradient of hexane/ethyl acetate and ethyl acetate/methanol. These processes afforded ten sub-fractions (Frs. H-V1 to H-V10). Compound **1** was obtained as crystals (1.11 g) from Fr. H-V4. Among the sub-fractions, the largest fraction, Fr. H-V2 (3.08 g), was applied to silica gel chromatography with hexane/chloroform (50:1), hexane/ethyl acetate (9:1 and 8:2), and then ethyl acetate/methanol (9:1 and 0:1) to yield seven fractions (Frs. H-V2-a to H-V2-g). Then, Fr. H-V2-b (1.85 g) was separated by preparative HPLC. Compounds **2** (9.7 mg) and **3** (53 mg) were obtained by elution with 70% aqueous acetonitrile; compounds **3** (5.9 mg) and **4** (3.2 mg) were obtained by elution with 85% aqueous methanol; compounds **5** (32.9 mg) and **6** (3.8 mg) were obtained by elution with 85% aqueous acetonitrile; and compound **7** (29.5 mg) was obtained by elution with 90% aqueous acetonitrile.

### 4.5. Cell Culture

Human acute leukemia cell line THP-1 and Jurkat cells were cultured in RPMI 1640 medium supplemented with 10% FBS and 2 mM glutamine. Cells were incubated at 37 °C under 5% CO_2_.

### 4.6. Determination of Cell Viability and Cell Death

WST-1 assay (Merck, Darmstadt, Germany) was used to determine cell viability following the manufacturer’s instructions. Cells were seeded in 96-well plates, cultured overnight, and treated with each of the compounds or etoposide (1 µM) as a positive control. After adding WST-1, cells were incubated for 4 h, and the absorbance was measured at 450 nm using a 96-well plate reader. Apoptotic cells were evaluated by flow cytometry using the APC Annexin V apoptosis detection kit with 7-AAD. Briefly, cells were seeded at a density of 1 × 10^5^/well in 24-well plates for 24 h, and then treated with garcinielliptone G (**5**) (10 μM) or various inhibitors, Z-VAD-FMK (50 μM), necrostatin-1 (10 µM), or ferrostatin-1 (1 µM), and incubated for 24 h. Cell suspensions were centrifuged and resuspended in 100 μL Annexin V binding buffer and stained with 5 μL of APC Annexin V and 5 μL of 7-AAD for 15 min each at room temperature. Stained cells were analyzed by FACSVerse. All examinations were carried out in triplicate.

### 4.7. Western Blot Analysis

Cell extracts were prepared in RIPA buffer containing 25 mM Tris/HCl pH 7.6, 150 mM NaCl, 1% Nonidet P-40, 1% sodium deoxycholate, 0.1% SDS and 1 × Complete™ protease inhibitor cocktail (Roche Diagnostics, Basel, Switzerland). The protein concentration was determined using the BCA protein assay kit (Thermo Fisher Scientific, Waltham, MA, USA). Lysates were heated at 95 °C for 5 min, subjected to SDS-PAGE, transferred to polyvinylidene difluoride transfer membranes, incubated with primary and secondary antibodies, and visualized using Amersham™ ECL™ Prime Western Blotting Reagent (Cytiva, Marlborough, MA, USA) and Immobilon Western Chemiluminescent HRP substrate (Millipore, Billerica, MA, USA), according to the manufacturer’s instructions. Primary antibodies used were anti-cleaved PARP, anti-cleaved caspase-3, anti-caspase-8, anti-caspase-9, anti-endonuclease G (EndoG) (1:1000; Cell Signaling Technology), anti-*β*-actin (1:10,000; Sigma Aldrich, St. Louis, MI, USA), and anti-H3 (1:1000; Millipore). Secondary antibodies were anti-rabbit HRP-conjugated antibody (1:10,000; Santa Cruz Biotechnology, Dallas, Texas, USA) and anti-mouse HRP-conjugated antibody (1:10,000; Santa Cruz Biotechnology).

### 4.8. Nuclear Extraction

THP-1 cells were seeded at a density of 1 × 10^6^ cells/well for 24 h prior to treatment. Following treatment with garcinielliptone G (**5**) (10 μM) at 37 °C for 24 h, intact nuclei were separated from the cytosolic fraction of THP-1 cells for further protein analysis using a nuclear extraction kit (FUJIFILM Wako Pure Chemical Corporation, Osaka, Japan). Differential centrifugation was performed according to the manufacturer’s protocol. Briefly, cells were collected, resuspended with the cytosol extraction reagent by vigorous vortex mixing for 30 min, and then centrifuged. The supernatant was collected as the cytosolic fraction and the pellet was collected as the nuclear fraction.

### 4.9. Statistical Analysis

All results are expressed as mean ± SD, calculated from the results of at least three independent experiments. Statistical significance was determined by analysis of variance (ANOVA) followed by the Tukey’s studentized range (HSD) test or one-way ANOVA with Dunnett’s multiple comparison test. *p* < 0.05 was considered statistically significant.

## 5. Conclusions

We have identified garcinielliptone G (**5**) as a cytotoxic principle of *Garcinia subelliptica* leaves by bioassay-guided isolation. Garcinielliptone G (**5**) showed growth inhibition by inducing apoptosis in human acute monocytic leukemia THP-1 cells and human T lymphocyte Jurkat cells. As garcinielliptone G (**5**) was found to induce apoptosis by both caspase-3 activation and a caspase-independent route, further detailed study of its apoptotic mechanism may provide information about garcinielliptone G (**5**) as a possible candidate for acute leukemia therapy.

## Data Availability

The data presented in this study are available within this article.

## Supplementary Materials

The following are available online, The supplementary material contains Figure S1: Cytotoxicity of extracts and fractions on THP-1 cell, Figure S2: Image analysis of apoptosis, Figure S3: Western blot indicated the nuclear translocation of EndoG, and Figure S4, Figure S10:^1^H-NMR spectra (500 MHz, CDCl_3_) of compounds (**1**–**7**).

## Sample Availability

Samples of the compounds are not available from the authors.
