# Supplementary figures and images for: Garcinielliptone G from Garcinia subelliptica Induces Apoptosis in Acute Leukemia Cells
Source: Molecules. 2021 Apr 21;26(9):2422. doi: 10.3390/molecules26092422 (PMC8122622; doi:10.3390/molecules26092422)

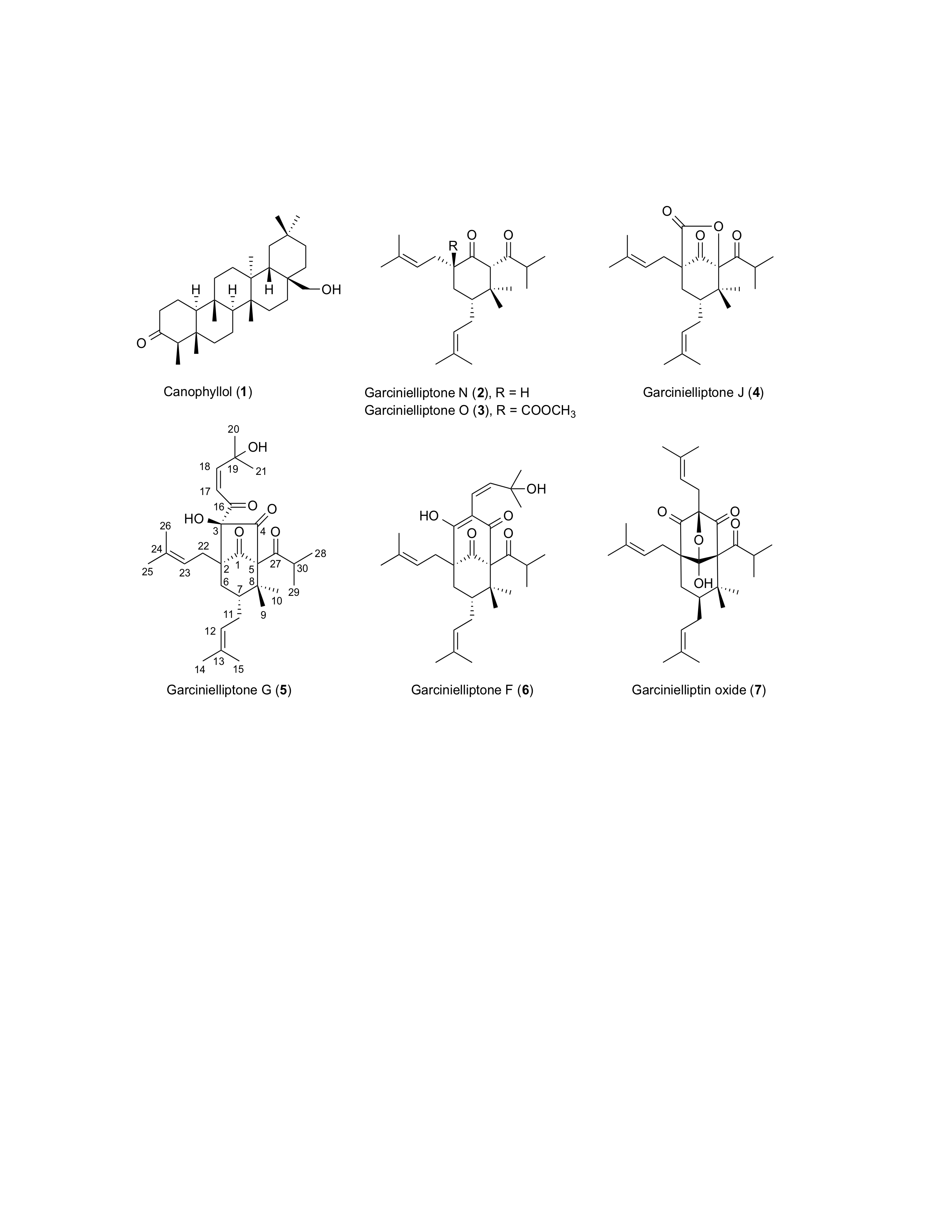

Supplement: Supplementary file 1 [file molecules-26-02422-s001.zip › Fig.1.tiff]

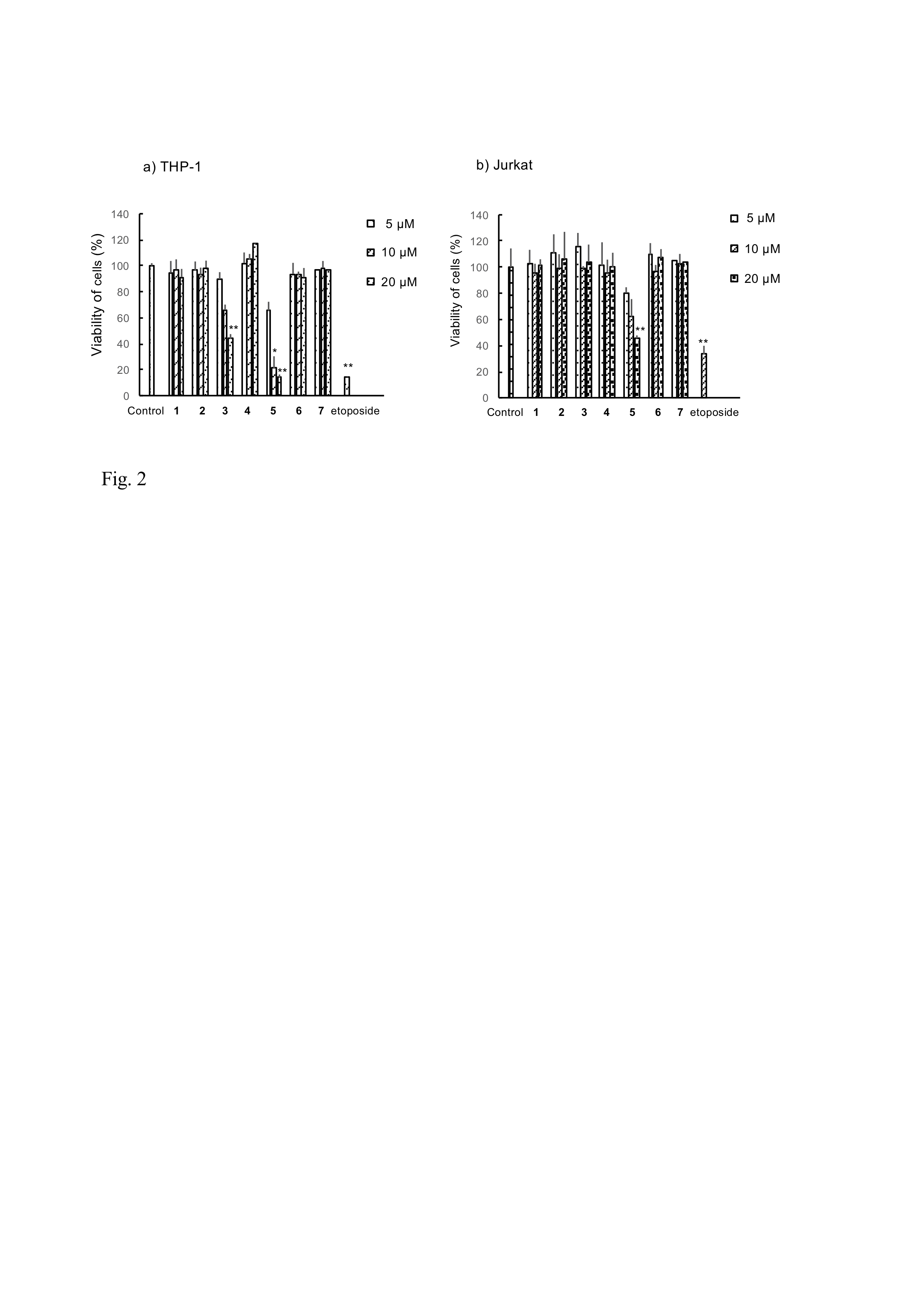

Supplement: Supplementary file 1 [file molecules-26-02422-s001.zip › Fig.2.tiff]

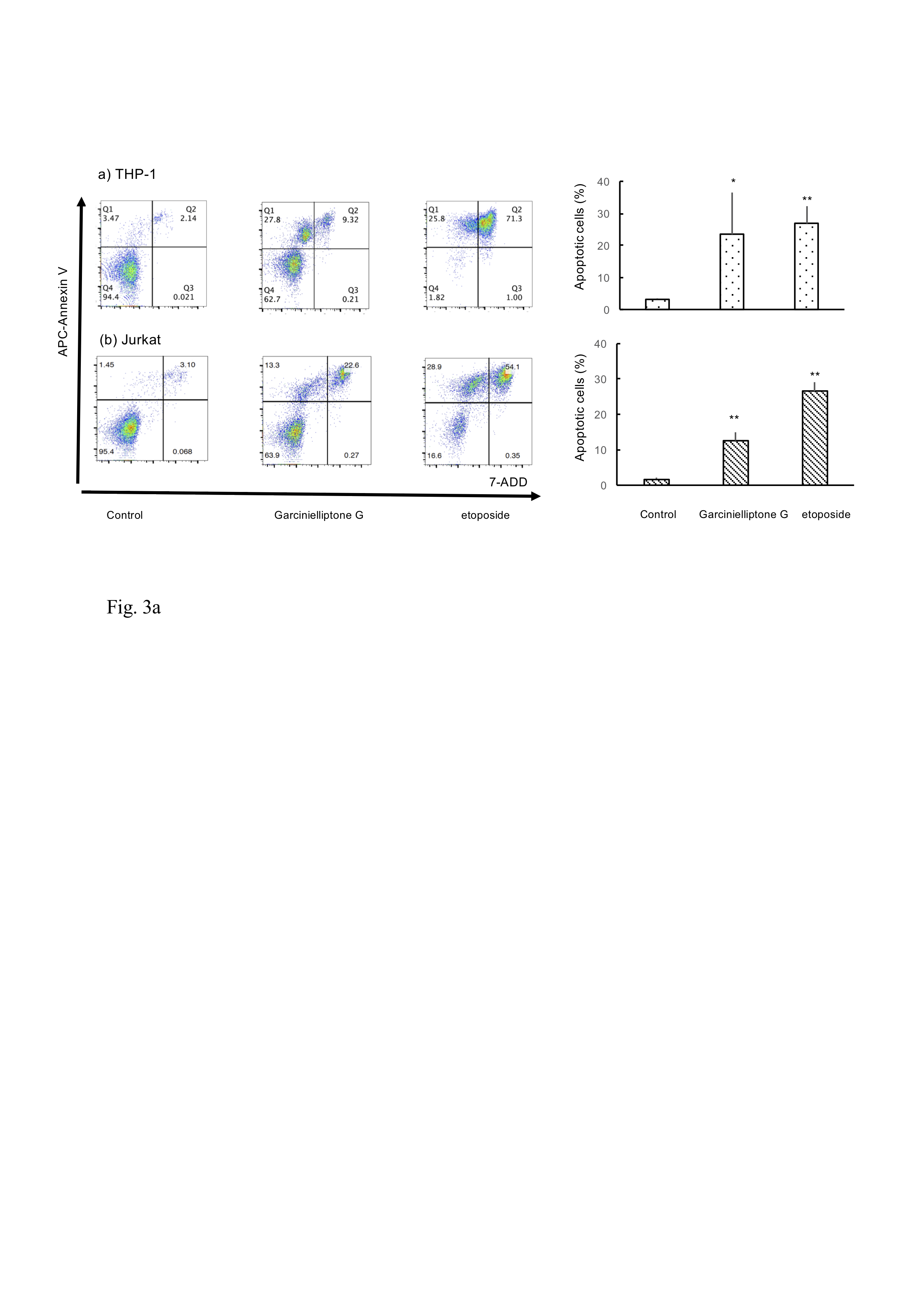

Supplement: Supplementary file 1 [file molecules-26-02422-s001.zip › Fig.3a.tiff]

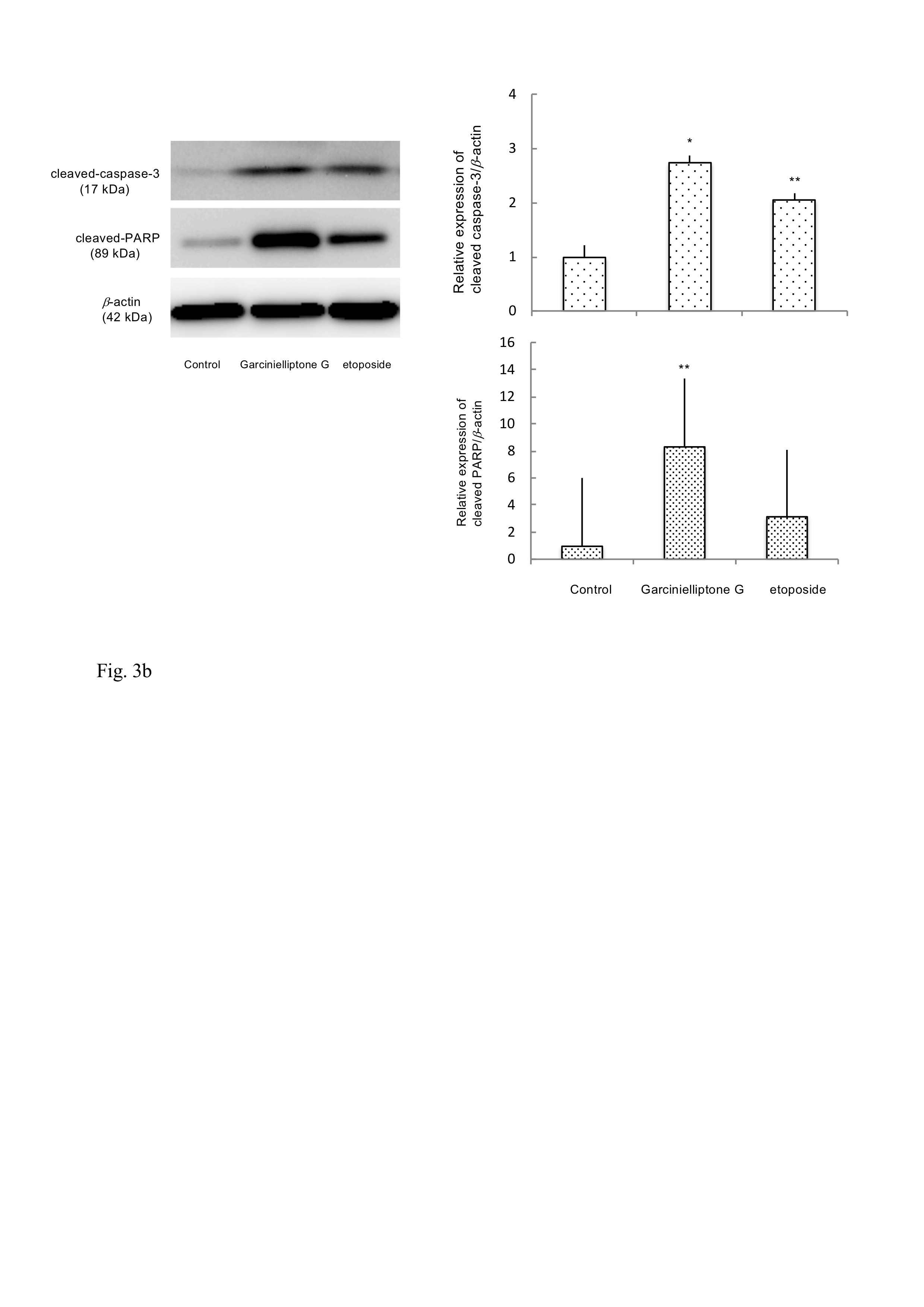

Supplement: Supplementary file 1 [file molecules-26-02422-s001.zip › Fig.3b.tiff]

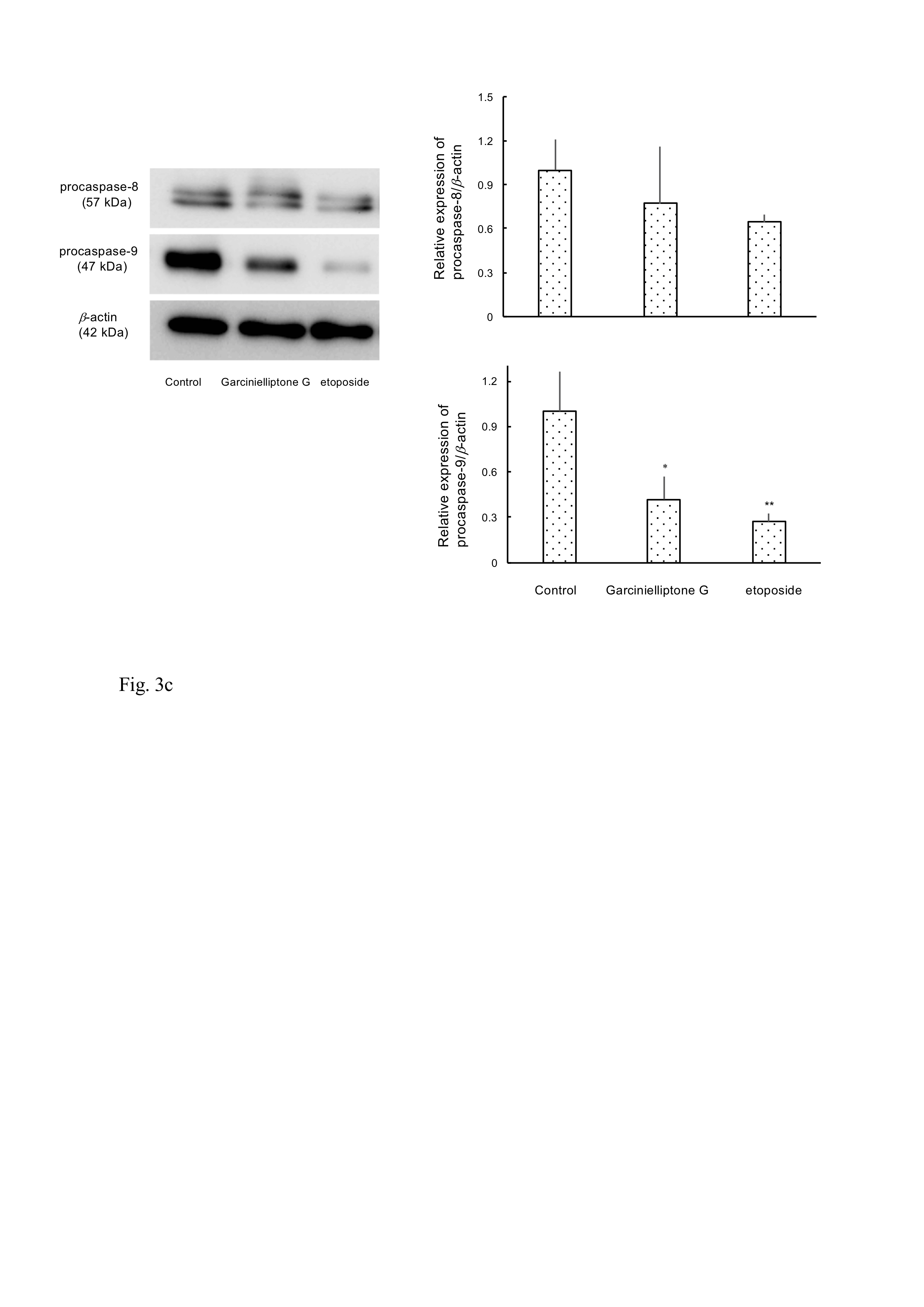

Supplement: Supplementary file 1 [file molecules-26-02422-s001.zip › Fig.3c.tiff]

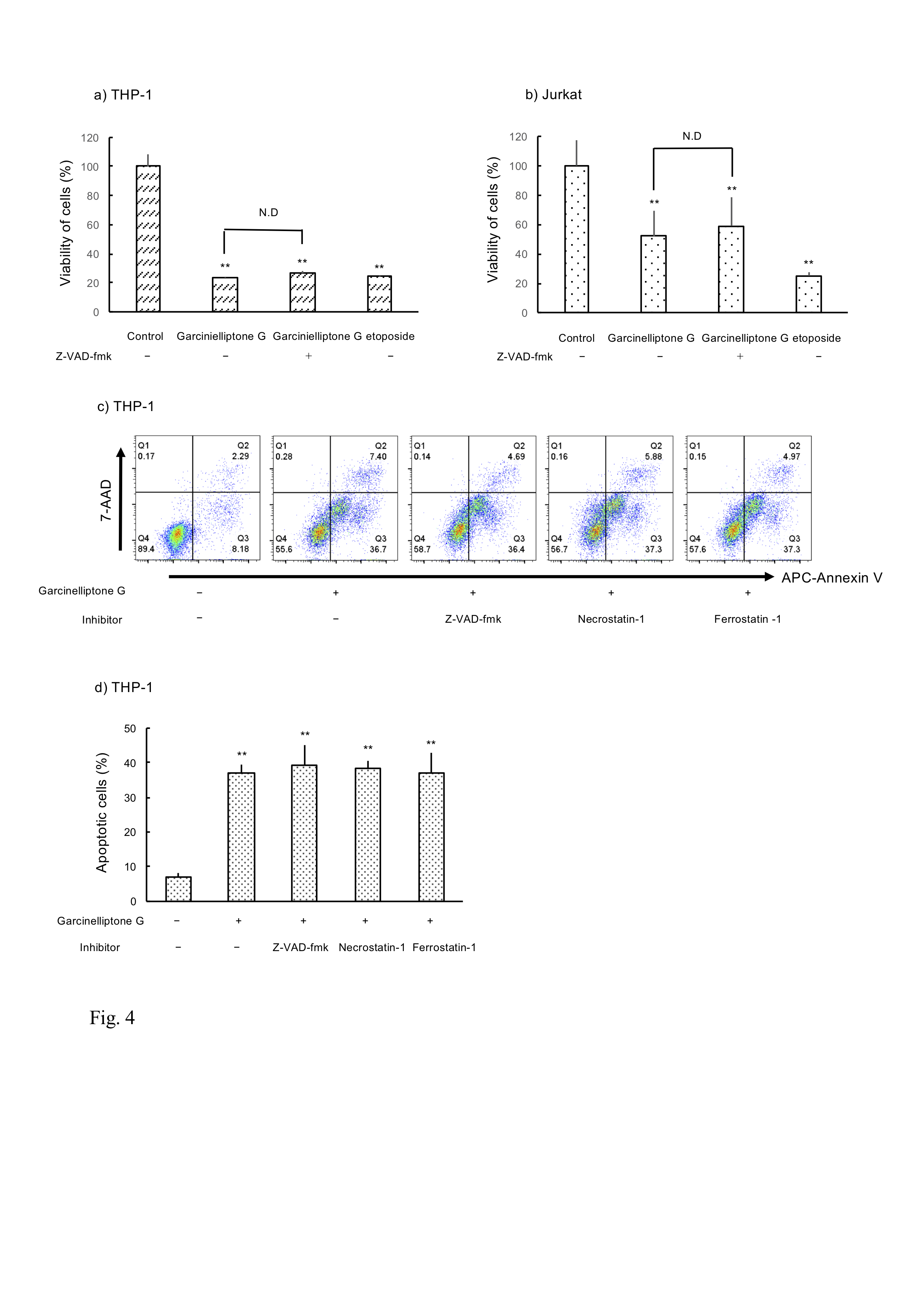

Supplement: Supplementary file 1 [file molecules-26-02422-s001.zip › Fig.4.tiff]
